# The complete chloroplast genome of *Primula odontocalyx,* a heterostylous species

**DOI:** 10.1080/23802359.2022.2135408

**Published:** 2022-11-15

**Authors:** Yunqi Liu, Li Zhang, Shubao Wang, Rui Li, Yuan Huang

**Affiliations:** School of Life Sciences, Yunnan Normal University, Kunming, P. R. China

**Keywords:** Chloroplast genome, *Primula odontocalyx*, phylogenomic analysis

## Abstract

*Primula odontocalyx* (Franch.) Pax (1905) is a perennial herb of the genus *Primula* in Primulaceae with heterostyly and ornamental value. Here, the chloroplast genome of *P. odontocalyx* was sequenced, assembled, and annotated. The complete chloroplast genome was a closed-circular molecule of 151,738 bp in length, containing a large single-copy region (LSC) of 83,817 bp, a small single-copy region (SSC) of 17,529 bp, and two inverted repeat (IR) regions of 25,196 bp. A total of 115 unique genes were annotated in the whole cp genome, including 81 protein-coding genes, 4 rRNA genes, and 30 tRNA genes. Phylogenetic analysis confirmed the close relationship between *P. odontocalyx* and *Primula moupinensis*, and both species belong to Sect. *Petiolares* Pax.

*Primula odontocalyx* (Franch.) Pax (1905) is a perennial herb (Figure 1) of Sect. *Petiolares* Pax in the genus *Primula* (Primulaceae) (Hu and Kelso 1996). *Primula odontocalyx* was placed in Sect. *Davidii* by Smith and Fletcher (1944). But now it was placed in Sect. *Petiolares* due to the absence of long hairs and basal bud scales at an early stage as shown by most specimens of this species (Richards 2003). *Primula odontocalyx* is mainly distributed in thickets and forest margins at an altitude of 900–3400 m in southern Gansu, western Hubei, southern Shanxi, Henan, and Sichuan Provinces of China (Hu and Kelso 1996). The flower of *P. odontocalyx* has 1–3 or rarely as many as 8 pink or lilac-rose corollas (Hu and Kelso 1996), this species, therefore, exhibits high ornamental value. In this study, we reported the first chloroplast genome of *P. odontocalyx*, which is useful for its taxonomic, systematics, and evolutionary studies.

**Figure 1.**
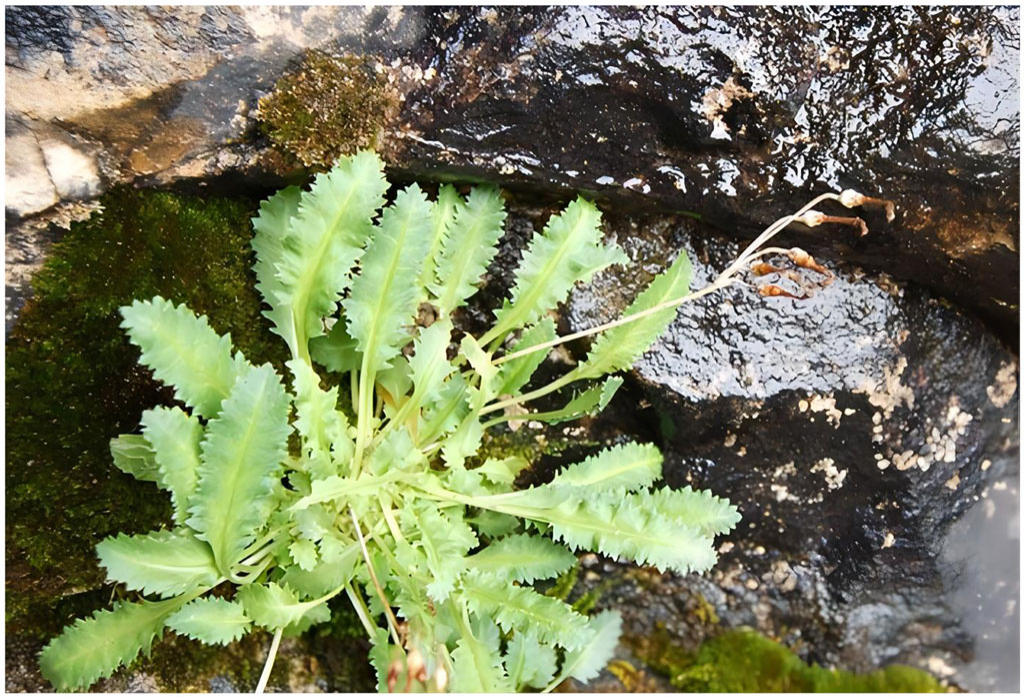
*Primula odontocalyx.* This image is provided by the corresponding author Yuan Huang, and is used with permission.

Fresh leaf of *P. odontocalyx* was collected from Chengkou of Sichuan Province, China (31.948°N, 108.665°E), and the corresponding voucher specimens were deposited in the Herbarium of Yunnan Normal University (Kunming, China; Jianlin Hang, hjlyuun@163.com) under the accession number of HY-24. Total genomic DNA was extracted using a modified CTAB method (Porebski et al. 1997) and 300 bp short-insert libraries were constructed following the manufacturer’s protocol (Illumina Inc., USA), and then paired-end sequenced on Illumina Hiseq X Ten sequencer platform. We obtained 14,914,454 filtered reads and then assembled the plastid genome using software NOVOPlasty v4.3.1 (Dierckxsens et al. 2017) with *Primula sinensis* Sabine ex Lindley as reference genome (GenBank accession No: NC_030609). The assembled genome was annotated using Geneious v2020.1.1 (Kearse et al. 2012), with *Primula moupinensis* (Genbank accession No. NC_050244) as the reference genome.

The complete plastid genome of *Primula chionantha* was assembled into a circular molecule with a length of 151,738 bp (Figure 2), average coverage of 506.5, and GC content of 37.0%, respectively, the NCBI accession number of the plastid genome is ON416872. The plastid genome of this species has a large single-copy region of 83,817 bp and a small single-copy region of 17,529 bp, which are separated by a pair of inverted repeats of 25,196 bp. In total, the plastid genome has 134 genes, 89 protein-coding genes (CDS), 37 tRNA genes, and 8 rRNA genes are annotated in the plastome, of which 115 genes, 81 protein-coding genes, 30 tRNA genes, and 4 rRNA genes are unique, respectively.

**Figure 2.**
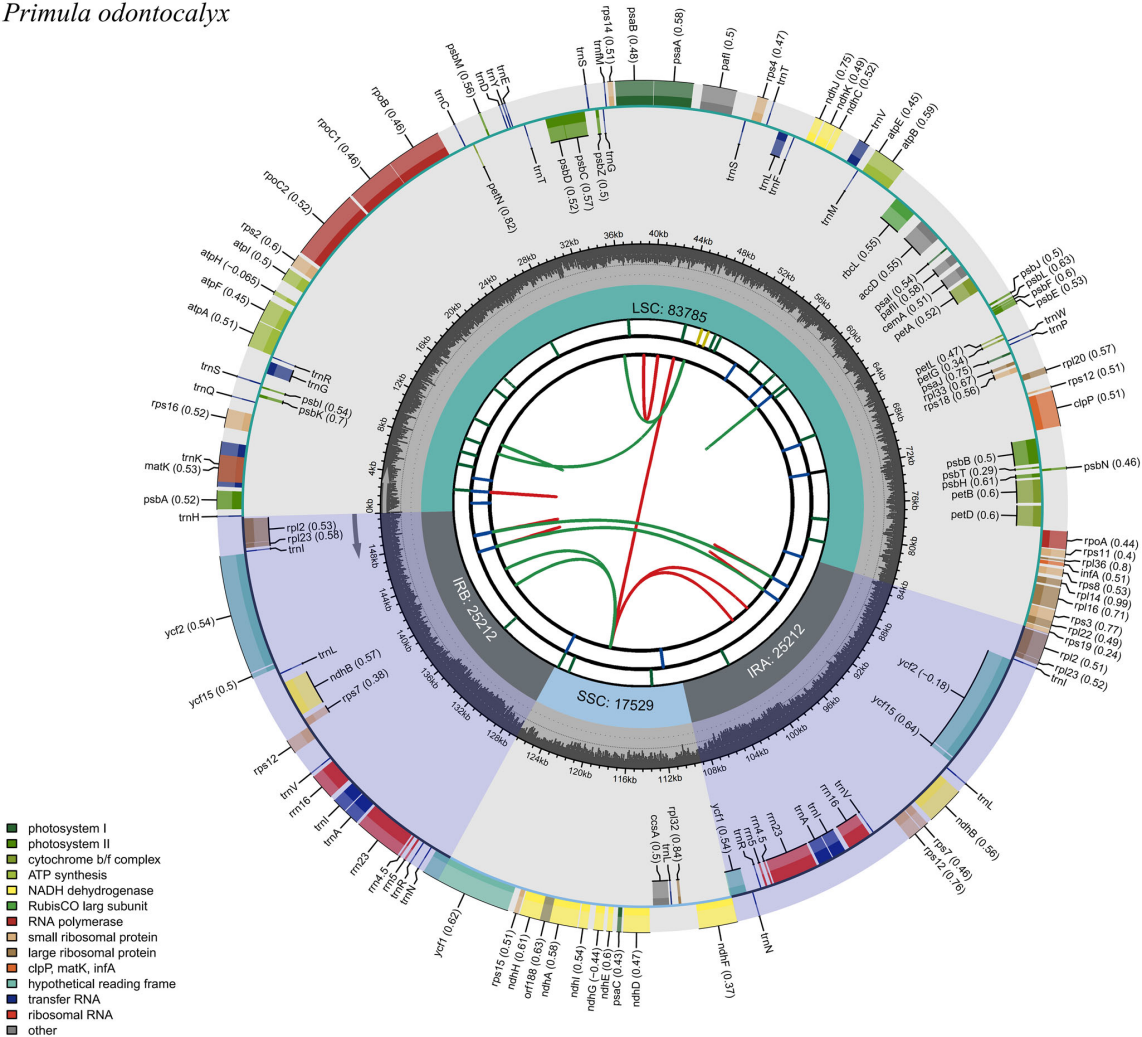
Genomic map of *Primula odontocalyx* chloroplast genome generated by CPGview. Genes drawn inside the circle are transcribed clockwise, and those outside are transcribed counterclockwise. Genes belonging to different functional groups are color-coded. The dashed area in the inner circle indicates the GC content of the chloroplast genome of *Primula odontocalyx.*

In order to further determine the phylogenetic position of *P. odontocalyx* within the genus *Primula*, we downloaded 39 *Primula* plastomes and two *Androsace* species, two *Lysimachia* species, and *Glaux maritima* (Liu et al. 2021) as outgroups. A total of 45 chloroplast genome sequences were aligned using MAFFT v7.47 (Katoh and Standley 2013). A maximum-likelihood phylogenetic tree was constructed using IQ-TREE v1.6.10 (Nguyen et al. 2015), and the best-fitted model is TVM + F+R2 using ModelFinder according to Bayesian information criterion (Kalyaanamoorthy et al. 2017). Branch supports were tested using ultrafast bootstrap (UFBoot) (Hoang et al. 2018) and SH-like approximate likelihood ratio test (SH-aLRT) (Guindon et al. 2010) with 10,000 replicates. The ML tree showed that *P. odontocalyx* was highly supported to be a sister species to *P. moupinensis*, forming a monophyletic clade with the rest species of Sect. *Petiolares* (Figure 3). This result supports that *P. odontocalyx* belongs to Sect. *Petiolares*, rather than Sect. *Davidii*. Additionally, the topologic structure of the phylogenetic tree showed that the relationships between *Primula*, *Androsace*, *Lysimachia*, and *Glaux* are consistent with previous phylogenetic studies (Mast et al. 2001).

**Figure 3.**
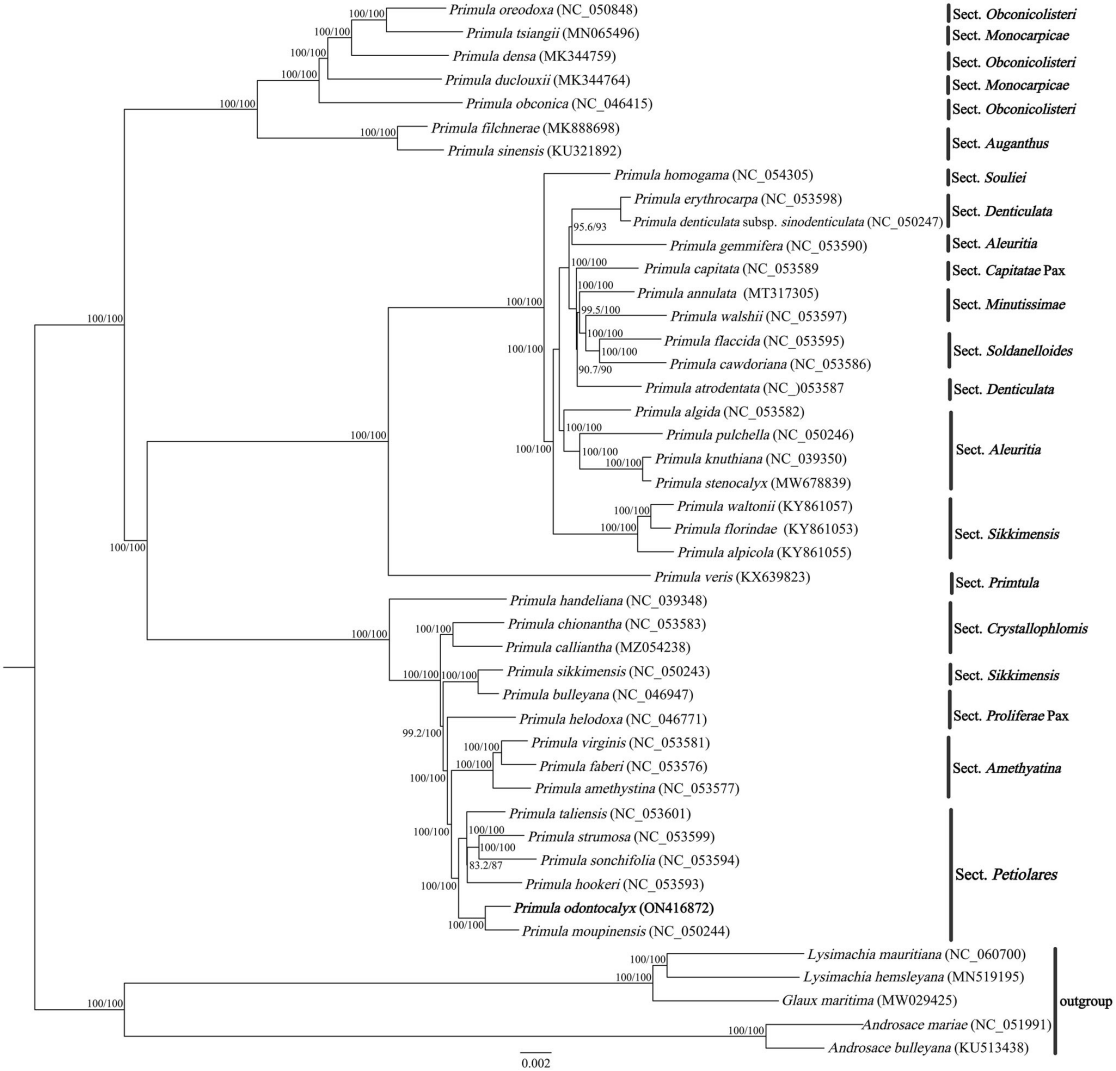
ML phylogenetic tree of *Primula odontocalyx* and 44 Primulaceae species based on chloroplast complete genomes, the branch supports values were reported as SH-aLRT/UFBoot.

## Supplementary Material

Supplemental Material

Supplemental Material

Supplemental Material

## Data Availability

The genome sequence data that support the findings of this study are openly available in GenBank of NCBI at [https://www.ncbi.nlm.nih.gov/] under accession no. ON416872. The associated BioProject, SRA, and Bio-Sample numbers are PRJNA834967, SRR19141931, and SAMN28088246, respectively.
