# Supplementary figures and images for: The complete chloroplast genome of Primula odontocalyx, a heterostylous species
Source: Mitochondrial DNA B Resour. 2022 Nov 15;7(11):1979–82. doi: 10.1080/23802359.2022.2135408 (PMC9673793; doi:10.1080/23802359.2022.2135408)

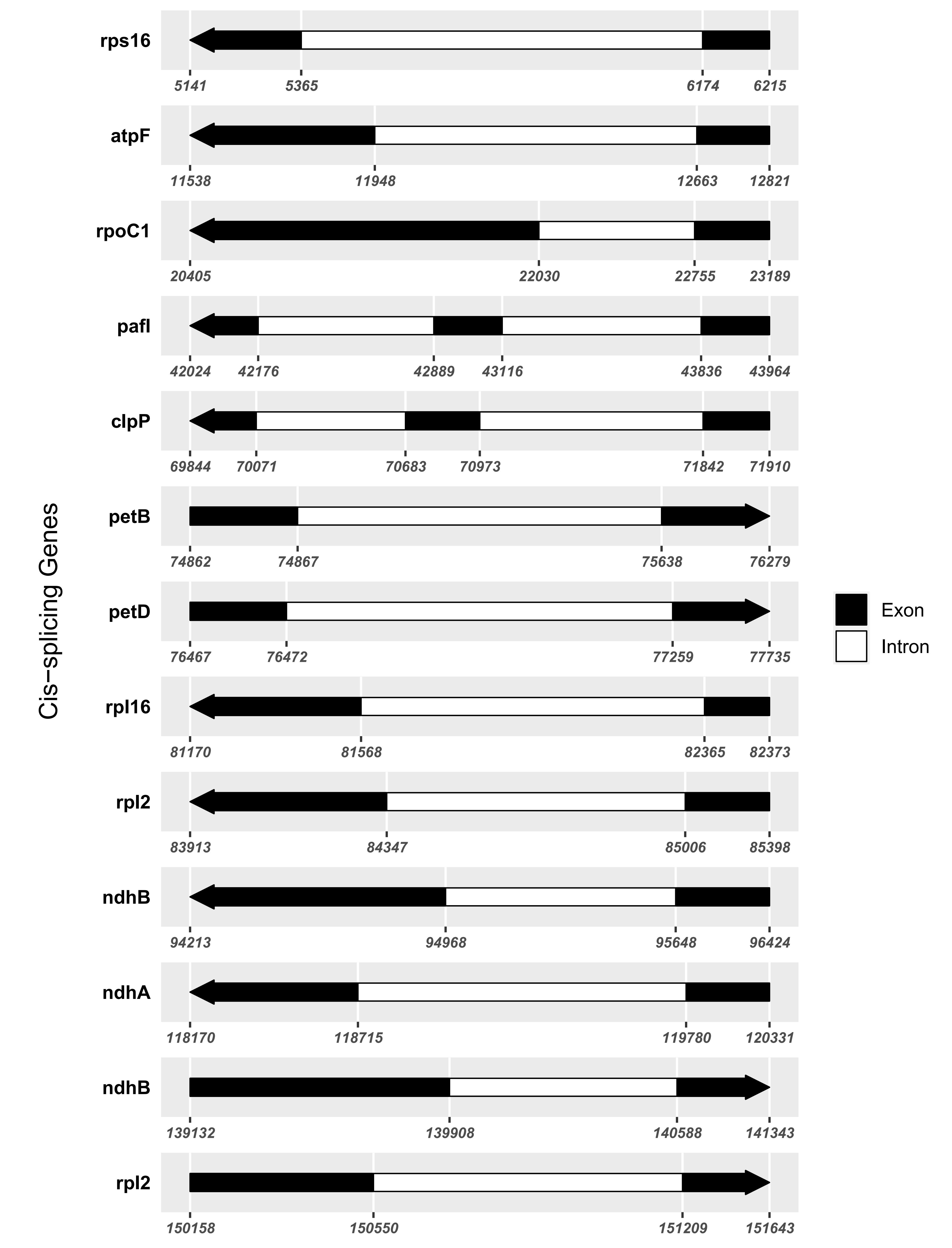

Supplement: Supplemental Material [file TMDN_A_2135408_SM6743.jpg]

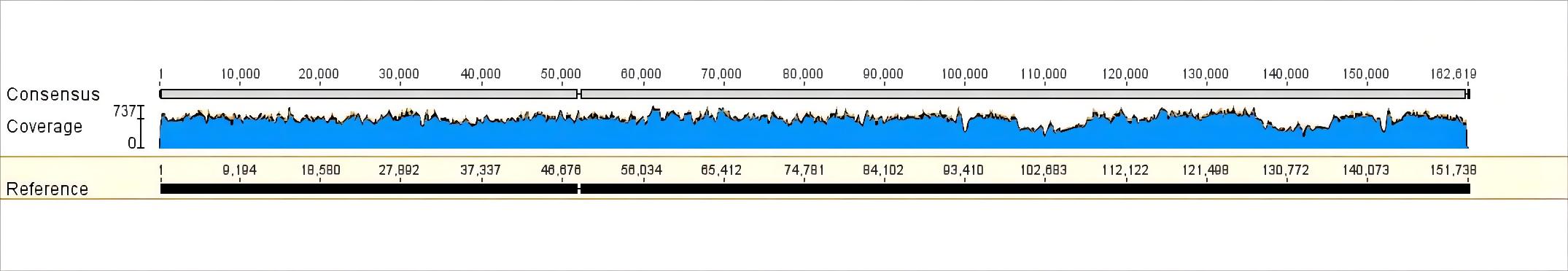

Supplement: Supplemental Material [file TMDN_A_2135408_SM6742.jpg]

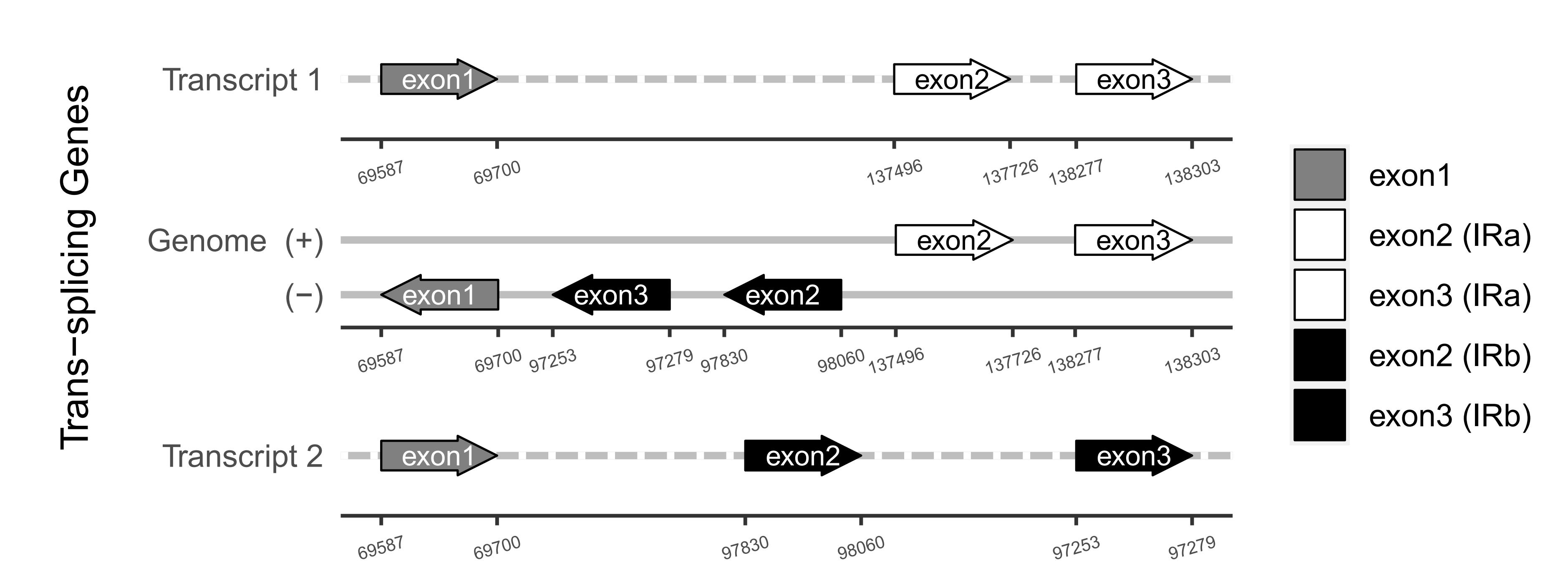

Supplement: Supplemental Material [file TMDN_A_2135408_SM6741.jpg]
